# 

**Published:** 1921-09-17

**Authors:** 


### One Physician to 726 People.

Contradicting a statement in the American newspapers that there is a shortage of 25,000 physicians in the U.S.A., the *Journal of the American Medical Association* says "there is no shortage of physicians in the United States." It states that, based on figures of the present Census, and on the figures in the last "American Medical Directory," 1921 (now in London), the United States has still one physician to every 726 people. Though there has been a reduction during the past fifteen years in the number of medical colleges, there is still an adequate quota to supply easily the number of physicians annually required. The problem is one of distribution.

### Passing Events and Topics.

### No Birth Registration Laws.

In the United States of America only twenty-three States have efficient birth registration laws; eighteen have imperfect ones; and five have none at all.

### Medicine in Emergencies.

Glasgow Corporation, with commendable municipal enterprise, has been investigating the facilities available for obtaining medicines from druggists' shops in the centre of the city at times when shops are ordinarily closed, and it has received a report from the medical officer of health with a list of druggists in various parts who undertake the dispensing of medicine outside ordinary business hours. The Corporation has now asked its medical officer of health to investigate and report upon the facilities available at hospitals, dispensaries, etc., for a supply of medicines.

### Medical Appointments.

University Intelligence.—The following appointments have been made in the Faculty of Medical Sciences at University of London, University College: Mr. R. A. Dart and Mr. J. Shellshear, to be senior demonstrators in anatomy; Dr. C. Da Fano, to be senior lecturer in histology; Dr. Elizabeth Fraser, to be senior assistant in zoology; Mr. E. B. Verney, to be assistant in physiology.

Dr. H. Sharpe, Tuberculosis Officer, Newcastle-under-Lyme, has been appointed Medical Superintendent of King George's Sanatorium for Sailors, Bramshott, Hants, a branch of the Seamen's Hospital Society.

September 17, 1921] [Price Twopence

### The Hospital

**RATES OF SUBSCRIPTION** (post free, payable in advance).

Three months, 3/3; six months, 6/6; twelve months, 13/-. (Should the cost of printing and paper as well as increased postage, necessitate alteration of these rates, the period paid for will be reduced proportionately on unexpired subscriptions.) **THE HOSPITAL "Index" to Volume LXIX., October 1920 to March 1921, is now ready, price 1/1 per copy, post free.** Address The Manager, THE HOSPITAL, "The Hospital" Building, 28/29 Southampton Street, Strand, London, W.C. 2.

